# Sick leave length and the costs of operatively and conservatively treated distal radius fractures in the working age population: a retrospective cohort study

**DOI:** 10.1186/s12891-023-06963-0

**Published:** 2023-10-25

**Authors:** Vili Palola, Teemu P. Hevonkorpi, Ville T. Ponkilainen, Antti P. Launonen, Ville M. Mattila

**Affiliations:** 1 Faculty of Medicine and Health Technology, Tampere University, Tampere, 33520 Finland; 2 Department of Surgery, Central Finland Central Hospital Nova, Jyvaskyla, Finland; 3 Department of Orthopedics and Traumatology, Tampere University Hospital, Tampere, Finland

**Keywords:** Distal radius fracture, Sick leave, Work, Costs, Treatment

## Abstract

**Background:**

Among the working population, a transient loss of working ability due to distal radius fracture (DRF) has a societal impact in terms of sick leave. Non-operative cast immobilization is the most common treatment option for DRF. However, these fractures are increasingly treated operatively. This retrospective cohort study of patients aged 20–64 with DRF compares the effects of different treatment strategies on sick leave length and overall cost of treatment.

**Methods:**

Multivariable regression analysis was used with treatment modality as an exposure and sick leave length as an outcome. Sick leave data were obtained from a national register. Costs were evaluated by adding the direct cost of the treatment modality to the mean cost of sick leave per patient in different treatment groups.

**Results:**

Of 614 working-age patients with a DRF who were treated at a tertiary hospital in Finland between January 2013 and December 2014, 521 were primarily treated non-operatively with cast immobilization and 93 were primarily operated. Of the primarily non-operatively treated patients, 48 were operated during follow-up. The mean follow-up was 5 years. The median time lost from work after DRF was 55 days (7.9 weeks), and the separated medians by treatment modality were 49 (7 weeks) and 70 days (10 weeks) for conservative and operative treatment, respectively. Multivariable linear regression analyses were performed for those patients who had sick leave (n = 292). Regression analysis also showed that operative treatment correlates with longer sick leave.

**Conclusions:**

Operative treatment of distal radius fracture led more often to longer time lost from work than conservative treatment. Moreover, due to longer sick leave and the costs of the operation itself, operative treatment is over two times more expensive than conservative treatment.

**Trial registration:**

retrospectively registered.

**Supplementary Information:**

The online version contains supplementary material available at 10.1186/s12891-023-06963-0.

## Background

Among the general working population, a transient loss of working ability due to a distal radius fracture (DRF) has a societal impact in terms of sick leave. Therefore, DRF can be considered a rising public health problem from the economic viewpoint. Keeping the loss of working ability due to DRF to a minimum is of interest to patients, employers, and the institutions responsible for paying sick leave benefits.

In the literature, there is a scarcity of knowledge on how treatment regimens affect sick leave length and time to return to work after a DRF [1]. For the treatment of DRF, the choice between cast immobilization and operative fixation of the fracture is greatly influenced by the radiographic assessment of the fracture, especially among working-age patients. Moreover, on some occasions, surgery is considered to enable an earlier return to normal function [1, 2]. This reflects well with the previous literature which has mainly focused on the association between radiological parameters and physical impairment in terms of grip strength, range of motion and, more recently, patient-reported outcome measures (PROMs) [3–7].

However, clear evidence of the radiographic thresholds of fracture malalignment that can lead to inferior functional outcomes after DRF is nonexistent at one year. In addition, long-term results only show a minor association between radiographs and functional outcomes [8, 9]. With more extreme values of radiographic malalignment, decreased functional outcomes have been reported [10, 11].

The primary objective of this study was to examine how different treatment strategies and patient baseline characteristics affect the duration of sick leave after a DRF. Additionally, the study evaluated the costs of each of the treatment strategies. Sick leave length and questionnaires regarding work and employee status were compared between patients treated with cast immobilization and those who underwent operative treatment. It was hypothesized that treatment modality, the physical demands of a job, the educational level of the patient, and clinical characteristics may have an association with sick leave length.

## Methods

### The registry data

This study was based on retrospective cohort of patients with DRF aged 20–64 years at the time of the injury who were treated at a tertiary hospital in Finland (Tampere University Hospital) between January 2013 and December 2014. The study protocol was approved by the Regional Ethics Committee of Tampere University Hospital (June 12, 2018, ETL-code R18103), and institutional approval was obtained from Tampere University Hospital research center (June 2018). After approving the protocol, we got permission to use the data and send surveys to patients. All patients recorded in the electronic health records with an International Classification of Diseases 10th edition (ICD-10) diagnosis code of S52.5 (distal radius fracture) or S52.6 (distal antebrachium fracture) during the study period were included to the study. Based on data from patient documents stored in the electronic health records, those patients with multiple traumas, inaccurate or incorrect DRF diagnosis, severe substance abuse, severe chronic disease, or patients with insufficient medical records (foreign citizens) were excluded from the study. In addition, patients who had died or sustained another DRF during the follow-up period were also excluded. Thus, study questionnaires were only sent to those patients aged 20 to 64 years with a confirmed isolated DRF. The study questionnaires were mailed to the patients 4 to 5 years after DRF. The questionnaires assessed the basic characteristics of work, educational level, handedness, and the physical demands of work. All patient details were de-identified for data analysis and manuscript writing, and all analyses were performed after protocol approval.

In countries with a strong social welfare system, employees who are injured have the right to sick leave benefits and employers must, by law, find substitute employees to perform the duties of the injured employee. In Finland, the Finnish Social Insurance Institution (SII) records the paid sick leave of employees lasting more than 10 working days. In the present study, the sick leave length was assessed from the SII register, which includes data on all permanent residents of Finland issued with a personal identity number. The personal identity number was then used to merge health records data with data on sick leave length. For those patients whose fracture was due to a work-related accident, there is a different system of compensation. All employers are obliged to take out insurance to cover occupational accidents and diseases from an insurance company. The Workers Compensation Center (WCC) collects data on the compensation paid by insurers to employers. Data on sick leave status and the compensation paid out by insurers to the patients with DRF was collected also from the WCC.

### Cost comparison

The costs of operative treatment versus conservative treatment was evaluated by adding the direct costs of the treatment modality to the mean cost of sick leave per patient in the different treatment groups. The costs of the treatment modality were evaluated using the Diagnosis Related Group (DRG) pricing system. The mean cost of sick leave per patient was calculated separately for each of the two treatment groups by multiplying the average daily sick leave cost by the mean number of working days lost. Finally, the costs of each of the treatment modalities were compared.

### Fracture classification

Ulnar variance, radio-ulnar inclination angle, dorsal angulation, and presence of intra-articular component of the fracture were evaluated from radiographs before and after closed reduction. Thereafter, the DRFs were classified into one of three groups: non-displaced, primarily stable, and primarily unstable. If the fracture did not require reduction at all, DRFs were classified as non-displaced. If the fracture required reduction and acceptable alignment was achieved, cast treatment was chosen and the fracture was classified as primarily stable. In cases of non-acceptable fracture reduction, operative treatment was chosen and the DRF was classified as primarily unstable.

### Statistics

The means, standard deviations (SD) and 95% confidence intervals (CI) were calculated for normally distributed variables. The median and interquartile range (IQR) were calculated for non-normally distributed variables. Mann Whitney U-test for independent samples was conducted to compare differences in median age between patients who had sick leave from work and those who had not. The categorical variables between the two compared groups were analyzed by Chi Square test. To compare the effect of treatment modality on sick leave length, a multivariable regression analysis was used with treatment modality as an exposure and sick leave length as an outcome. Two separate multivariable analyses were performed for those groups who had sick leave and those who returned the questionnaires. For multivariable analysis including patients with sick leave data, seven patients were excluded as outliers in the analysis. For another analysis included only those patients who returned the questionnaires, four patients were excluded (two patients were outliers in the analysis and two had missing data on handedness). Collinearity between treatment modality and fracture classification were checked and the model was accepted (variance inflation factor, VIF < 2). The covariates for the multivariable regression analysis were chosen by using a directed acyclic graph (DAG) [12], which is presented in Appendix 1.

In the multivariable analysis, the following six variables were included: age, sex, classification of the fracture, educational level of the patient, physical demands of the job, and whether the fractured hand was dominant. DAGs have been used to investigate causal relationships that involve multiple interrelated variables. In addition, DAG is useful for avoiding bias in variable selection and for identifying confounding.

## Results

### Baseline characteristics

**Fig. 1:**
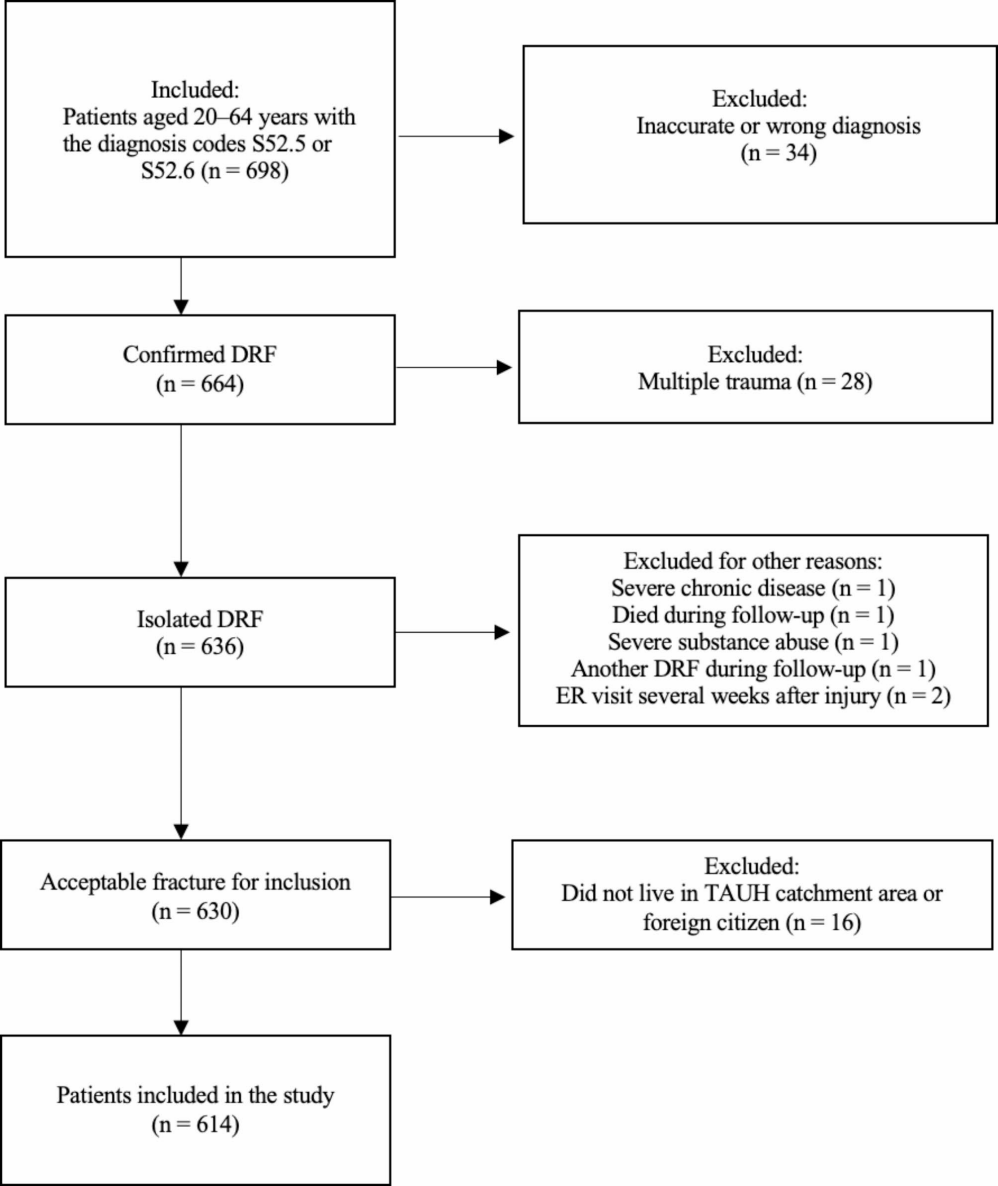
A flow chart of patient inclusion and exclusion criteria

Between January 2013 and December 2014, a total of 698 working-age patients who had sustained a DRF were treated at Tampere University Hospital (TAUH). Of these, 68 patients had either multiple traumas, mistakenly coded DRF diagnoses, or other detailed reason and were excluded. In addition, patients who did not live in the catchment area of the University Hospital or were not Finnish citizens were excluded (n = 16). A flow chart of patient inclusion and exclusion criteria is presented in Fig. 1.

In total, 614 patients aged 20–64 years with DRF were included in the study. Most of the patients were women (n = 403; 66% women and n = 211; 34% men). The median age of the patients was 51.0 years (IQR 36–58). Cast immobilization was chosen primarily for 521 (85%) patients and operative treatment for 93 (15%) patients. The most common surgical technique was open reduction and internal fixation (ORIF) using volar locking plates (VLP) (90% of all operatively treated patients). Of the primarily non-operatively treated patients, 48 patients suffered loss of reduction during cast treatment and were converted to operative treatment. There were 42 operations with VLP, one external fixation, four corrective osteotomies, and one revascularization of os trapezius due to post-traumatic osteonecrosis. The mean follow-up was 5 years.

### Sick leave

A sick leave of more than ten days was observed in 292 (48%) patients (208 women, 84 men). Of these patients, the gender-specific median ages were 54 years in women and 41 years in men. No age difference was observed between those patients who had not had sick leave from work and those who had (median ages 50 vs. 51years; p = 0.93). Interestingly, a greater percentage of women had sick leave after DRF than men (52% in women and 40% in men; p = 0.01). The median time lost from work after DRF was 55 days (IQR 42–76). The treatment comparison and baseline characteristics of those patients who had sick leave of more than ten days are listed in Table 1.

**Table 1:** The treatment comparison and baseline characteristics of patients who had sick leave of more than ten days

Baseline Characteristics of Patients (sick leave > 10 days)	All (n = 292)	Cast immobilization (n = 212)	Operative treatment (n = 80)
Age, median (IQR)	51.0 (38.0–58.0)	52.0 (37.0–58.0)	50.0 (42.5–56.8)
Female, n (%)	208 (71)	153 (72)	55 (69)
Male, n (%)	84 (29)	59 (28)	25 (31)
Fracture classification			
“Non-displaced”, n (%)	55 (19)	all	
“Primarily stable”, n (%)	186 (64)	157	29
“Primarily unstable”, n (%)	51 (17)		all
Duration of sick leave in days, median (IQR)	55 (42–76)	49 (40–67)	70 (57–114)

In total, 127 (43%) out of 292 patients completed and returned the study questionnaires (Table 2). There were 17 (13%) non-displaced and 90 (71%) primarily stable fractures. During the cast treatment, 18 of these 90 (20%) fractures were classified as a secondarily unstable and treated operatively afterwards

**Table 2:** The treatment comparison and baseline characteristics of those patients who returned the completed questionnaires

Patients who returned the questionnaires	All (n = 127)	Cast immobilization (n = 89)	Operative treatment (n = 38)
Age, median (IQR)	51.0 (38.0–58.0)	51.0 (36.0–58.0)	50.5 (46.0–57.0)
Female, n (%)	97 (76)	70 (79)	27 (71)
Male, n (%)	30 (24)	19 (21)	11 (29)
Dominant hand injury*, n (%)	62 (49)	43 (48)	19 (50)
Educational Level			
Basic level (9 years), n (%)	23 (18)	15 (17)	8 (21)
Secondary education (12 years), n (%)	59 (46)	45 (51)	14 (37)
College education (13 + years), n (%)	45 (35)	29 (32)	16 (42)
Employees			
Physical demand of job, yes (%)	70 (55)	49 (55)	21 (55)
Physical demand of job, no (%)	43 (34)	33 (37)	10 (26)
Physical demand of job, moderate/varied (%)	14 (11)	7 (8)	7 (18)
Work-related injury, n (%)	23 (18)	13 (15)	10 (26)

* two patients did not report their handedness

For multivariable analysis including 292 patients, the model resulted in sick leave 24.5 (CI 8.7–40.2) days longer for patients who had undergone operative treatment than for those who had conservative treatment when adjusted for age, sex, and classification of fracture. The model suggests that these four variables can explain 10.2% of the variation in sick leave duration (R2 = 0.10). When only those patients who returned the questionnaires (n = 127) were included, the multivariable linear regression model adjusted for age, sex, classification of fracture, educational level, physical demands of work, and whether the fractured hand was dominant resulted in sick leave 24.3 (CI 4.6–43.9) days longer for operative treatment than for conservative treatment. When questionnaires were included in the multivariable analysis, the model explained 23.0% of the variation in sick leave duration (R2 = 0.23).

### Cost comparison

In Finland, the cost of a single day of sick leave is estimated to be €370 [13]. In the present study, the mean cost of sick leave was €10,745 per patient in the conservatively treated patient group, and €23,029 in the operatively treated group. In addition, based on the DRG prices for different surgical methods, we calculated the average cost of a surgical operation to be €1599, whereas the DRG price for non-operative cast immobilization is €317. In summary, when combining these costs, we found that operative treatment (€24,628) was on average €13,566 (123%) more expensive than conservative treatment (€11,062).

## Discussion

The main finding of the present study was that the operative treatment of DRF more often resulted in longer sick leave and absence from work than conservative treatment. Furthermore, we found that only 48% of patients had sick leave of more than ten days, and therefore had the right to sick leave benefits after DRF. Interestingly, paid sick leave was more common in female patients than in male patients.

In our study, the median time lost from work after DRF was 55 days (7.9 weeks), and the separated medians by treatment modality were 49 (7 weeks) and 70 days (10 weeks) for conservative and operative treatment, respectively. In previous studies, the median sick leave length has varied between 4 and 8 weeks [14, 15]. A study by MacDermid et al. found the average sick leave from work among 227 workers with DRF to be 9.2 weeks (median 8) and the strongest predictors for sick leave were reported to be self-reported disability and occupational demands [14]. However, 21% of workers did not have sick leave from work and 10% lost only one or two days. In addition, they concluded that work loss after DRF cannot be accurately predicted based solely on clinical variables [14]. This is in line with our findings that the treatment of a DRF only explains a small part of the variation in the length of sick leave, i.e. factors other than those considered in our model have a strong influence. In another study, Egund et al., who studied professionally active men aged 20–65, reported that the median time lost from the work after DRF was 4 weeks and that almost a third of patients did not require any sick leave [15]. In addition, they reported that sick leave length differed between conservatively and operatively treated patients (median 4 vs. 8), which is in line with our findings.

Although we had a greater number of weeks away from work in our study, the results are comparable to the findings reported in previous studies. The weakness of these previous studies was that their sick leave data were self-reported, which may not agree with administrative data on work loss and may lack accuracy and include recall bias [16]. Another possible explanation for our findings regarding the greater number of weeks away from work is that our data contained information on patients who had sick leave of more than ten days. As a result, the calculated mean values of absence from work in our study were higher compared to those of other studies. Finally, differences in the social insurance systems of different countries could also have affected the time lost from work.

Interestingly, we found the operative treatment of DRF resulted in longer time lost from work than conservative treatment. This was against the hypothesis that operative treatment should speed up recovery after DRF and lead to an earlier return to previous level of work. Ax et al. also reported that operative treatment leads to longer sick leave than conservative treatment [17]. This finding might, in part, be related to injury severity, including more complicated fracture morphology and more severe soft tissue injuries. Another possible explanation is psychosocial factors. When an injury requires surgery, the patient may feel entitled to a longer period of sick leave due to the seriousness of the injury, although earlier studies have reported superior short-term functional outcomes with operative treatment [2, 18]. Therefore, doctors should encourage patients to return to work early after DRF surgery if patients feel they are capable of doing so, even if sick leave length had initially been estimated to be longer.

Perhaps the most unexpected finding in this study was that only 292 of 614 patients received sick pay during their leave of absence. There are several possible explanations for this finding. First, employment rates of people aged 20–64 in Finland were 73.3% in 2013 and 73.1% in 2014. Hence, our study population also included people who were unemployed. Second, there are no national registers that record sick leave periods of less than 10 days. Therefore, data on those who took shorter sick leave periods could not be included in the present study.

On average, the direct cost of conservative treatment with cast immobilization after distal radius fracture is substantially less than the cost of operative treatment [19]. Although the cost of operative treatment in different countries can vary widely, this study showed that operative treatment is over two times more expensive than conservative treatment due to longer sick leave and the costs of the operation itself. However, we were not able to calculate all the costs related to DRFs, and the episode-based indirect costs associated with this injury may have been underestimated. In particular, the number of physical therapy contacts and additional visits to the hospital are missing from our data.

A key strength of the present study was the accuracy of the SII register that includes information on absence from work, with practically no missing information. All sick leave episodes after DRF were medically certified, eliminating self-report bias. In addition, this study comprises one of the largest published cohorts of working-age patients with a single injury (DRF). However, the present study also has some limitations. First, there are no national registers that record sick leave periods of less than 10 days. The lack of this information may have affected the mean numbers of time loss from work. Moreover, due to the limitations of the database, we could not demonstrate the total number of patients who required sick leave. Secondly, the cohort is relatively small for follow-up and there is also the risk for recall bias. Although recall bias is always a possible in self-reported studies, we consider its importance to be small risk in this study, since information on working place at the time of fracture and the physical demands of the job are rather easy to remember. With a high probability, the patients also remember whether the fracture happened at work. Further, we consider the level of education and the handedness mentioned by the patient to be reliable information, because they do not change over time.

## Conclusions

The main finding of the present study was that the operative treatment of distal radius fracture led more often to longer time lost from work than conservative treatment. Due to longer sick leave and the costs of the operation itself, operative treatment is over two times more expensive than conservative treatment from an economic point of view. Furthermore, approximately half of the working-age patients in the present study had more than a ten-day absence from work after distal radius fracture, and thus had the right to sick leave benefits. Further research is required to improve the treatment guidelines for distal radius fracture in the working-age population.

### Electronic supplementary material

Below is the link to the electronic supplementary material.

Supplementary Material 1

## Data Availability

The datasets generated and/or analysed during the current study are not publicly available due to privacy policy, based on ethical approval and Finnish legislation, but are available from the corresponding author on reasonable request.

## Abbreviations

DRF: Distal radius fracture
ICD-10: International Classification of Diseases 10^th^ edition
SII: Finnish Social Insurance Institution
WCC: Workers Compensation Center
DRG: Diagnosis Related Group
SD: Standard deviation
CI: 95% confidence interval
IQR: Interquartile range
DAG: Directed acyclic graph
ORIF: Open reduction and internal fixation
VLP: Volar locking plate
TAUH: Tampere University Hospital
SGML: Standard
