# Supplementary material for: Sick leave length and the costs of operatively and conservatively treated distal radius fractures in the working age population: a retrospective cohort study
Source: BMC Musculoskelet Disord. 2023 Oct 25;24:842. doi: 10.1186/s12891-023-06963-0 (PMC10601330; doi:10.1186/s12891-023-06963-0)
Supplement: Supplementary file 1 — Supplementary Material 1 [file 12891_2023_6963_MOESM1_ESM.docx]

**Appendices**


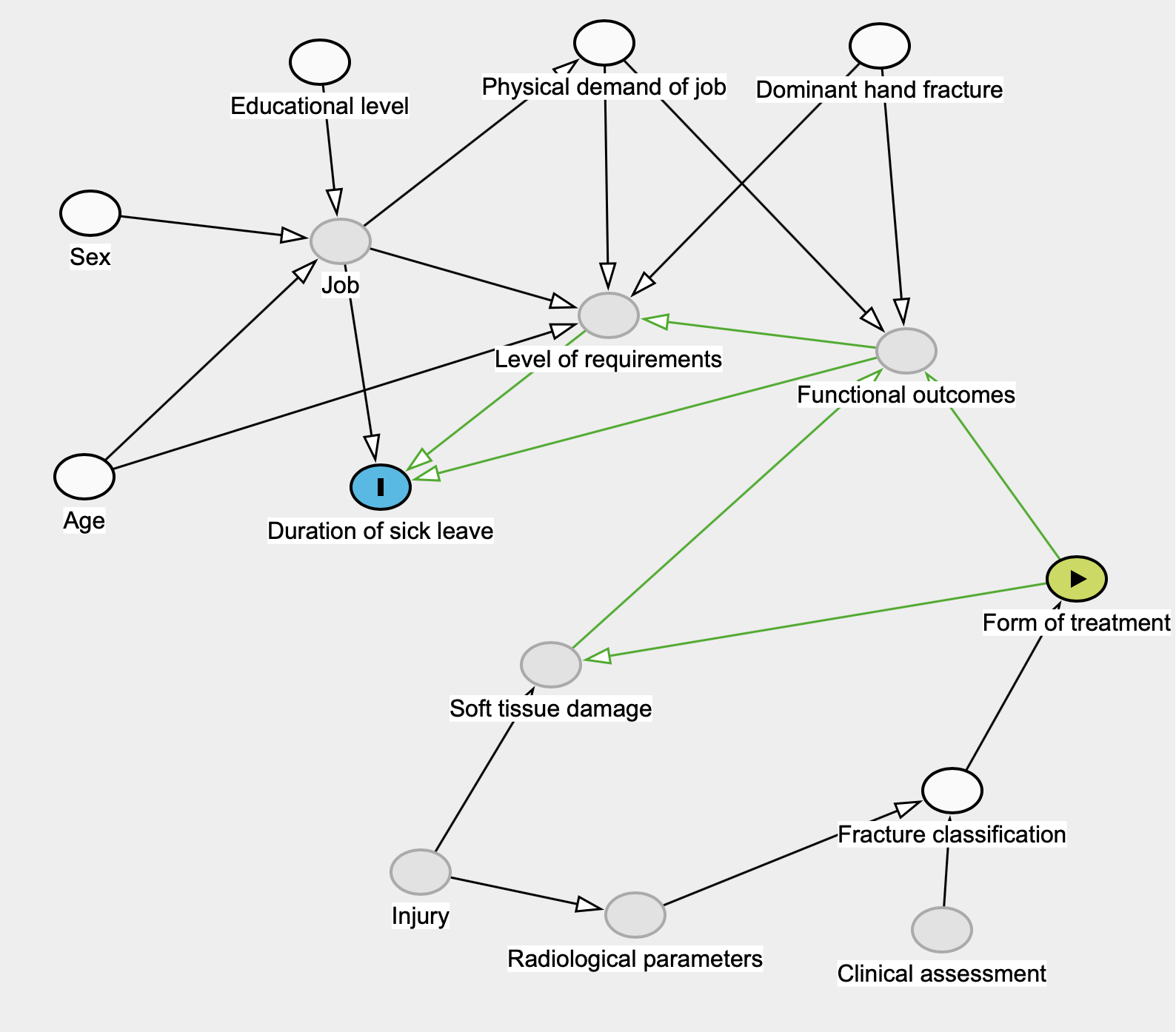


*Appendix 1. Directed acyclic graph (DAG) representing the causal relationships behind the multivariable model investigating the effect of type of treatment on sick leave length.*
